# Induction and persistence of radiation-induced DNA damage is more pronounced in young animals than in old animals

**DOI:** 10.18632/aging.100340

**Published:** 2011-06-18

**Authors:** Darryl Hudson, Igor Kovalchuk, Igor Koturbash, Bryan Kolb, Olga A. Martin, Olga Kovalchuk

**Affiliations:** ^1^ Department of Biological Sciences, University of Lethbridge, Lethbridge, AB, T1K 3M4 Canada; ^2^ Department of Neuroscience, University of Lethbridge, Lethbridge, AB, T1K 3M4 Canada; ^3^ Laboratory of Molecular Pharmacology, Center for Cancer Research, NCI, NIH, Bethesda, MD, 20892, USA; ^4^ Current address: Division of Biochemical Toxicology, National Center for Toxicological Research, US Food and Drug Administration. AR 72079 USA; ^5^ Current address: Department of Radiation Oncology, Peter MacCallum Cancer Centre, East Melbourne, VIC, 8006 Australia

**Keywords:** radiation, DNA damage and repair, aging

## Abstract

Younger individuals are more prone to develop cancer upon ionizing radiation (IR) exposure. Radiation-induced tumors are associated with inefficient repair of IR-induced DNA damage and genome instability. Phosphorylation of histone H2AX (γ-H2AX) is the initial event in repair of IR-induced DNA damage on the chromatin flanking the DNA strand breaks. This step is crucially important for the repair of DNA strand breaks and for the maintenance of genome stability. We studied the molecular underpinnings of the age-related IR effects using an animal model. By assaying for IR-induced γ-H2AX foci we analyzed the induction and repair of the DNA strand breaks in spleen, thymus, liver, lung, kidney, cerebellum, hippocampus, frontal cortex and olfactory bulb of 7, 14, 24, 30 and 45 days old male and female mice as a function of age. We demonstrate that tissues of younger animals are much more susceptible to IR-induced DNA damage. Younger animals exhibited higher levels of γ-H2AX formation which partially correlated with cellular proliferation and expression of DNA repair proteins. Induction and persistence of γ-H2AX foci was the highest in lymphoid organs (thymus and spleen) of 7 and 14 day old mice. The lowest focal induction was seen in lung and brain of young animals. The mechanisms of cell and tissue-specificity of in vivo IR responses need to be further dissected. This study provides a roadmap for the future analyses of DNA damage and repair induction in young individuals.

## INTRODUCTION

Ionizing radiation (IR) is capable of inducing DNA damage such as cross linking, nucleotide base damage, and DNA single and double strand breaks (SSBs and DSBs) [1-3]. DSBs are the most dangerous lesions. If unrepaired or misrepaired, they may lead to genome instability and carcinogenesis. IR exposure results in elevated rates of blood malignancies [4-11], breast cancer [6, 12-14], thyroid cancer [4, 6], stomach and lung cancers [6], bladder cancer [15], and renal-cell carcinomas [16]. Extensive epidemiological data show that radiation-induced cancer incidence is the highest in the exposed children [17-20]. This IR-induced DNA damage is the only well-established risk factor for childhood cancers [21]. Similar to humans, radiation exposure causes lymphoma, leukemia, liver, breast and kidney tumors in mice [22-30]. Furthermore, the lifetime cancer incidence and mortality is highest when mice are irradiated during the neonatal and puberty period [25, 27-32]. The molecular mechanisms of the age-related predisposition to radiation-induced cancer are not well understood [33-34].

In response to endogenous and genotoxic stress-induced DNA damage, cells orchestrate a complex network of repair processes [33, 35]. The initial event is phosphorylation of a H2A histone family member H2AX at serine 139 (forming γ-H2AX) in the chromatin flanking the DNA double-stranded ends [36-37] which form nuclear foci at DSB sites [38]. γ-H2AX is crucially important for the repair of DNA DSBs and for the maintenance of genome stability [39-40]. A direct correlation has been found between H2AX phosphorylation and the number of DSBs resulting from radiation [41-43]. Therefore, γ-H2AX foci are used as efficient biomarkers of DNA damage and repair [36]. Animal studies are well recognized as invaluable tools to dissect the mechanisms of *in vivo* IR responses. We set out to dissect the molecular underpinnings of the age-related radiation effects using a well-established mouse model. We hypothesized that altered ability to deal with IR-induced damage may be seen in young individuals during the period of active growth. By assaying for the levels of IR-induced γ-H2AX foci we analyzed the induction and repair of the IR-induced DNA DSBs in spleen, thymus, liver, lung, kidney, cerebellum, hippocampus, frontal cortex and olfactory bulb of 7, 14, 24, 30 and 45 days old male and female mice as a function of age.

Here we systematically studied induction and persistence of IR-induced γ-H2AX in animals tissues as a function of animal age. We also show that γ-H2AX focus incidences partially correlate with cellular proliferation and expression of DNA repair proteins.

## RESULTS AND DISCUSSION

Five mouse organs and four mouse brain regions were examined and compared in very young (7 and 14 days old), adolescent (24 days old), young adult (30 days old) and sexually mature adult (45 days old) male and female mice for the incidence of γ-H2AX focus induction and persistence after exposure to 1 Gy of X rays. The maximum formation of γ-H2AX foci was analyzed 30 minutes post-exposure, while persistent responses were studies 24 hours post-IR [43-44].

### Radiation-induced generation of γH2AX foci in somatic tissues of young, adolescent and mature mice

**Figure 1:**
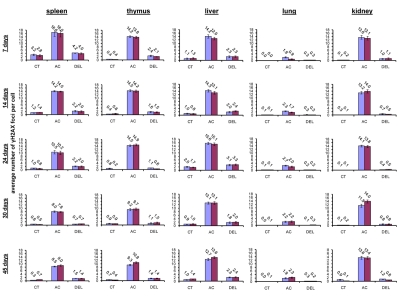
Radiation-induced DNA damage and cell proliferation in somatic tissues of male and female mice of different ages. Incidences of γ-H2AX foci in spleen, thymus, liver, lung and kidney tissues of 7, 14, 24, 30 and 45 days old sham-irradiated and 1 Gy irradiated male and female mice. Data are presented as average number of γ-H2AX foci per cell. CT-control, AC- acute effect, 30 minutes after exposure; DEL-delayed effect, 24 hours after exposure.

In somatic organs of the unexposed young 7 and 14 days old animals, the highest background levels of γ-H2AX foci were seen in spleen (3.2±0.4 foci per nucleus in 7 days old males, 2.8±0.5 - in 7 days old females, 1.2±0.2 - in 14 days old males and 1.4±0.2 foci per cell in 14 days old females), while the lowest levels were seen in lung (0 foci per nucleus in 7 days old males and females, 0.1±0.1 foci per nucleus in 14 days old males and 0.1 ±0.0 foci per nucleus in 14 days old females) and kidney tissue (0.1±0.1 foci per nucleus in 7 days old males, 0.2±0.1 - in 7 days old females, 0.3±0.1 - in 14 days old males and 0.1±0.0 foci per cell in 14 days old females) (Fig. 1).

**Figure 2:**
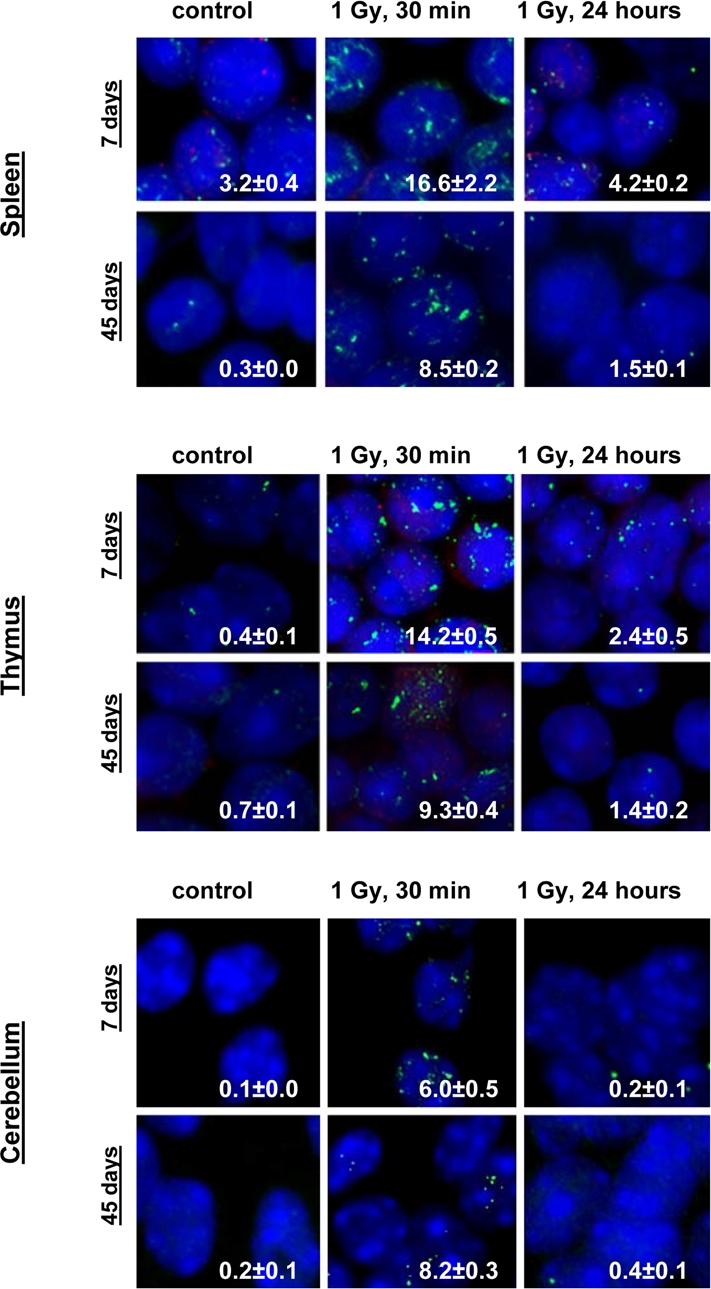
Representative immunostaining of γ-H2AX in murine tissues. Representative images showing the presence of γ-H2AX foci in spleen, thymus and cerebellum tissues of 7 and 45 days old male mice irradiated with 0 or 1 Gy of X-rays. Green, γ-H2AX; blue, DNA stained with DAPI. Average numbers of foci per cell? standard error are shown in the right bottom corner of the images (100x magnification).

Exposure to 1 Gy of X-rays caused significant DNA damage which was evidenced by a profound increase of the γ-H2AX foci levels in all the somatic tissues of mice (Fig. 1). The increase in the number of IR-induced γ-H2AX foci was the highest in lymphoid organs (thymus and spleen) of 7 and 14 day old mice 30 minutes after exposure (in thymus - up to 14.2±0.5 foci per nucleus in 7 days old males, 13.8±0.6 - in 7 days old females, 14.5±0.5 - in 14 days old males and up to 14.2±0.6 foci per cell in 14 days old females; in spleen - up to 16.6±2.2 - in 7 days old males, 16.0±2.0 foci per cell in 7 days old females, 14.2±0.3 - in 14 days old males and up to 14.0±0.3 foci per cell in 14 days old females) (immunostaining is shown in Fig. 2). The lowest focal induction levels were seen in lung tissues of 7 days old animals (up to 1.9±0.4 - in 7 days old males and up to 0.9±0.3 foci per cell in 7 days old females) (Fig. 1A). In older animals (30 and 45 day old mice) the induction of γ-H2AX focus levels was lower than in young animals (Fig. 2).

Almost all DNA damage in all the tissues analyzed was effectively repaired 24 hours after exposure, evident by the decrease of the γ-H2AX foci to near control levels (Fig. 1). Yet, some significant residual persistence of the IR-induced foci was seen in spleen, thymus, liver, lung and kidney of 7 and 14 days old animals.

The highest H2AX focal persistence was seen in thymus and spleen of 7 day old animals 24 hours after exposure (in thymus - 2.4±0.5 foci per nucleus in 7 days old males, 2.1±0.2 - in 7 days old females; in spleen - 4.2±0.2 foci per nucleus in 14 days old males, 4.0±0.4 - in 14 days old females) (Fig. 1). It was previously shown that animals exposed to IR at the age of 7 days exhibited significantly elevated levels of lymphoid tissue malignancies [25, 29]. Indeed, thymus and spleen are important targets for radiation carcinogenesis and recent studies have shown that elevated γ-H2AX levels are often found in onco-transformed cells [36, 45-47]. Thus, γ-H2AX persistence may be an important sign of predisposition to carcinogenesis [36].

The levels of induction and persistence of IR-induced γ-H2AX foci can also be related to the proliferative capacity of the tissue. It has been shown that cycling cells, especially S-phase cells are the most sensitive to IR-induced DNA damage compared to cells in other phases of the cell cycle [48-50].

**Figure 3:**
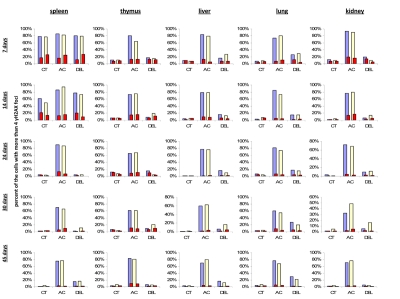
Levels of DNA damage and cell proliferation in spleen, thymus, liver, lung and kidney tissues of 7, 14, 24, 30 and 45 days old sham-irradiated and 1 Gy-irradiated male and female mice. Data are presented as a percentage of cells with more than 4 γH2AX foci per cell. Red bars represent the percentage of PCNA-positive cells that harbor more than 4 γH2AX foci per cell. CT-control, AC- acute effect, 30 minutes after exposure; DEL-delayed effect, 24 hours after exposure.

To check this hypothesis, we conducted double staining of the exposed and control mouse tissues for both γ-H2AX and the proliferating cells nuclear antigen (PCNA), a marker of S-phase cells [51-52]. We analyzed the percent of cells that harbor more than four γ-H2AX foci and are also positive PCNA-positive (Fig. 3). We concluded that in young animals there was a significant amount of cells that had persistent γ-H2AX foci 24 hours post-IR, yet this persistence was not just the result of cellular division. Though PCNA-positive cells consistently contain more γ-H2AX foci whether irradiated or not, exclusion of these cells from the data does not change the trend.

It has been suggested that the persistence of γ-H2AX foci seen 24 hours after exposure may be a critical factor determining cell survival [53]. While their exact properties are obscure, they can be attributed to un-repairable DSBs, incomplete or stalled repair of more complex DSB lesions, faulty rejoining of DSBs, lethal DNA lesions, persistent chromatin alterations, apoptosis, activity of several kinases and phosphatases, and checkpoint signaling [53-56, 57]. In the light of these findings, the molecular mechanisms and biological significance of the residual persistence of γ-H2AX foci in the tissues of young animals deserves special attention in the future.

We did not see any sex differences in the γ-H2AX focal induction in somatic tissues of male and female mice, with the exception of the lung tissue where the foci induction was 2 times higher in males than in females.). IR is a known risk factor for lung cancer [58-59] and we have previously shown that IR leads to strong and persistent induction of DSBs in male lung [60]. Furthermore, it is well established that lung cancer is much more prevalent in males [61]. Therefore, the molecular mechanism and biological repercussions of the sex differences in IR-induced γ-H2AX foci formation in the lung tissue will be dissected in future studies.

### Radiation-induced generation of γ-H2AX foci in brain of young, adolescent and adult mice

**Figure 4:**
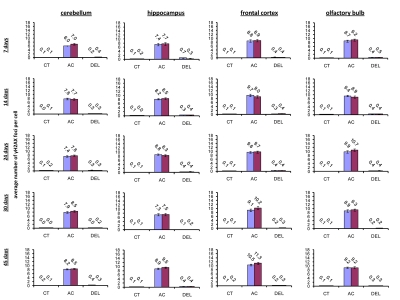
Radiation-induced DNA damage and cell proliferation in brain regions of male and female mice of different ages. Incidence of γ-H2AX foci in cerebellum, hippocampus, frontal cortex and olfactory bulb tissues of 7, 14, 24, 30 and 45 days old sham-irradiated and 1 Gy-irradiated male and female mice. Data are presented as average number of γH2AX foci per cell. CT-control, AC- acute effect, 30 minutes after exposure; DEL-delayed effect, 24 hours after exposure.

**Figure 5:**
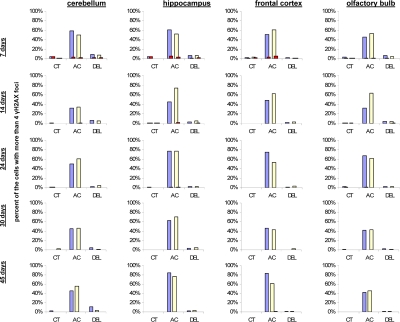
Levels of DNA damage and cell proliferation in cerebellum, hippocampus, frontal cortex and olfactory bulb tissues of 7, 14, 24, 30 and 45 days old sham-irradiated and 1Gy-irradiated male and female mice. Data are presented as a percentage of cells with more than 4 γH2AX foci per cell. Red bars represent the percentage of PCNA-positive cells that harbor more than 4 γH2AX foci per cell. CT-control, AC- acute effect, 30 minutes after exposure; DEL-delayed effect, 24 hours after exposure.

IR exposure also caused DNA damage in brain of exposed mice. We observed a very significant accumulation of 1 Gy of IR-induced γ-H2AX foci in all the studied regions (from 0.1-0.2 foci per nucleus in the un-irradiated brain tissues up to 6-10 foci per nucleus 30 minutes after exposure) (Fig. 4 and example of immunostaining in cerebellum is shown in Fig. 2). The majority of DSBs in brain were effectively repaired, and only slight residual persistence of γ-H2AX foci was noted 24 hours after exposure (in the range of 0.3-0.7 foci per nucleus on average) (Fig. 4 and cerebellum in Fig. 2). This persistence was the highest in the 7 days old male mice, especially, in their hippocampi (0.7±0.2 foci per nucleus). Hippocampus, a site of active neurogenesis in young animals is crucially important in memory and cognition. Therefore, elevated γ-H2AX foci persistence in hippocampus may be partially related to active neurogenesis. However, on the whole, focal persistence in brain was similar to the somatic tissues and only partially related to the increased proliferative capacity, since cellular PCNA levels were slightly increased only in the brain regions of 7 day old animals (Fig. 5).

Overall, somatic tissues were much more susceptible to radiation exposure than any brain regions (Fig. 1-5).

### DNA repair in young, adolescent and adult animals

**Figure 6:**
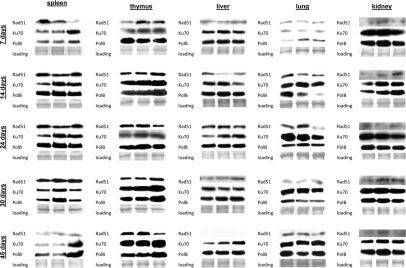
DNA repair in the somatic tissues of young, adolescent and adult animals. Western analysis of Ku70, Rad 51 and Polβ proteins. Representative blots from two independent technical repeats are shown; each experiment included cohorts of five animals for each exposure condition, with equal representation of each animal. Each lane represents pooled lysates from five animals.

IR exposure activates a battery of DNA repair mechanisms that are crucial to maintain the genome integrity [62-63]. To establish the correlation between the induction and repair of DNA damage and the DNA repair mechanisms, we measured the levels of proteins involved in DNA repair and those responsible for elimination of radiation-induced damage: homologous recombination (HR), non-homologous end joining (NHEJ) and base excision repair (BER) in somatic tissues and brain regions of control and radiation exposed mice. Specifically, we analyzed changes in Rad51, a key player involved in DSB repair via HR. This protein forms a nucleoprotein filament on single stranded DNA regions and catalyses the search for homologous sequences, strand paring and strand exchange [64]. We also studied expression of two other DNA repair proteins Ku70 and Polβ. Ku70 is a key participant in the NHEJ pathway to DSB repair [65-66]. Polβ plays a pivotal role in BER mechanisms which the cells employ to remove oxidized bases produced in access upon IR exposure [65-67]. It is known that BER intermediates, such as abasic sites and stand breaks, activate HR and NHEJ [67] in a process that involves H2AX phosphorylation. Persistence and efficiency of DNA repair may be linked to the cellular levels of the aforementioned proteins. We found that in somatic tissues Ku70 levels were the highest of all proteins, both in the control and exposed animals (Fig. 6). This observation is consistent with the fact that NHEJ is the prevalent mechanism of DNA repair and Ku70 is therefore the most abundant repair protein.

In spleen of 7 day old animals we observed induction of Ku70 and Polβ only 24 hours after exposure. This was consistent with the highest amount of persistent DNA strand breaks in the spleen of 7 days old mice. In 14, 24, and 30 day old animals Ku70 and Polβ induction was noted 30 minutes after exposure. Interestingly, in spleen we did not see any induction of Rad51 levels by IR. Furthermore, the cellular Rad51 levels in spleen decreased with age. In thymus of 7 day old mice, we found a significant induction of Rad51 30 minutes after exposure. In the other age groups only slight Rad51 induction occurred 24 hours after irradiation (Fig. 6). Ku70 was induced 30 minutes post-IR in thymus of young animals, and 24 hours post-IR of more mature ones. In liver and kidney, we saw slight increases in Ku70 and Polβ in the exposed animals of all age groups, except the very young ones. The levels of Rad51 were also very low, and further diminished with age.

In lung, we have not seen any strong up-regulation of DNA repair proteins in 14-45 day old animals. Furthermore, in the very young 7 day old animals the levels of DNA repair proteins were very low, and the significant induction was noted only in the Polβ levels. This may in turn partially explain the persistence of γ-H2AX foci in the lung tissue of exposed 7 day old animals (Fig. 6).

**Figure 7:**
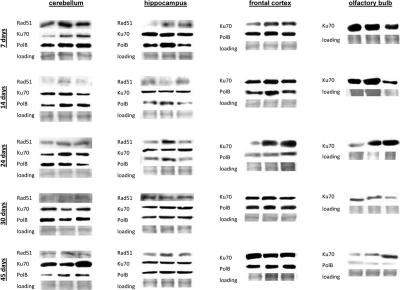
DNA repair in the brain tissues of young, adolescent and adult animals. Western analysis of Ku70, Rad 51 and Polβ proteins. Representative blots from two independent technical repeats are shown; each experiment included cohorts of five animals for each exposure condition, with equal representation of each animal. Each lane represents pooled lysates from five animals.

In brain, we found high levels of Ku70 and Polβ. Rad 51 was below detection levels in frontal cortex and olfactory bulb (Fig. 7). Yet, the high levels of Ku70 and Polβ and significant radiation inducibility of these proteins was enough to efficiently eradicate all the damage (Fig. 7). In the future it will be important to scrutinize the DNA repair fidelity as a function of both age and actual proteins activity.

### Conclusions and outlook

Our data show that tissues of young animals are much more susceptible to IR-induced DNA damage. Further, results indicate that younger animals have higher inducibility of repair proteins, whereas adult animals have higher steady-state levels. Overall, induction and persistence of DNA damage during the period of active growth may interfere with cellular programming and development, and therefore predispose younger individual to various toxic radiation effects, including tumors and cognitive deficits. The higher induction and persistence of γ-H2AX foci in the young animals, specifically, in their lymphoid tissue may lead to increased genome instability and consequently to higher levels of lymphoma and leukemia. Indeed, it has been shown that younger animals are more susceptible to IR induced tumors. Higher levels of DNA damage-induced γ-H2AX foci in brain of young animals may result in toxic radiation effects on brain, changes in memory and cognition, and even lead to increased frequency of brain tumors. Human data indicate that childhood IR exposure results in significantly higher rates of leukemias and brain tumors as well as memory and learning deficits.

Furthermore, exposure to diagnostic irradiation also poses a significant threat to young children [68]. As reported by Brenner and Hall [68], among all age groups, radiation-induced tumor risks are the highest in children and decrease with age. Growing children are much more radiosensitive, because they have a larger proportion of rapidly dividing cells [68].

While comparing IR responses of somatic organs and brain regions we noted that brain areas have the lowest basal levels of γ-H2AX and the highest repair potential, as seen by the resolution of the γ-H2AX foci. Even though some somatic organs have higher expression of repair proteins, brain tissues may have higher repair fidelity. From the organisms' point of view, this is highly likely, since mis-repair and apoptosis of brain permanent deleterious effects, whereas damaged somatic cells may undergo apoptosis and be replaced. Though the mechanisms of cell and tissue-specificity of *in vivo* IR responses need to be further dissected, our results validate the usefulness of these approaches for deciphering the underlying mechanisms behind the processes of IR-induced DNA damage repair and apoptosis. In the future it would be important to analyze the roles of p53 pathway, chromatin modifications and telomeres and telomere-associated proteins in the age- and tissue-specificity of radiation responses [33, 69-72].

Even though further animal studies are clearly needed before these data can be extrapolated to humans, this study provides a roadmap for the future analyses of DNA damage and repair induction in young individuals.

## MATERIALS AND METHODS

### Model and irradiation of animals

In this study, we examined DNA damage in somatic and brain tissues of young, adolescent and adult male and female mice following in vivo radiation exposure. Very young (7 and 14 days old), adolescent (24 days old), young adult (30 days old) and sexually mature adult (45 days old) male and female mice (30 animals/sex/age group) were randomly assigned to different treatment groups. Handling and care of animals was in strict accordance with the recommendations of the Canadian Council for Animal Care and Use (1993). The procedures have been approved by the University of Lethbridge Animal Welfare Committee. Animals were housed in a virus-free facility and given food and water ad libitum. The exposed cohort (20 animals/sex/age group) received 1Gy (2cGy/s) of X-rays (90 kV, 5 mA). Control mice (10 animals/sex/age group) were sham treated. All animals were humanely killed 30 minutes or 24 hours after exposure. The experiment was reproduced once using 8 animals sex/age group/treatment. The spleen, thymus, liver, lung, kidney, cerebellum, hippocampus, frontal cortex and olfactory bulb tissue were sampled upon sacrificed and processed for further molecular and cellular studies.

### Immunocytochemistry

The levels of radiation-induced damage were studied by accumulation of phosphorylated histone H2AX (γ-H2AX) foci. Studied tissues of control and experimental animals were touch-printed onto positively charged slides and processed for γ-H2AX immunohistochemistry using anti-γ-H2AX primary antibodies, as described [40, 56, 73]. For double-labeling primary rabbit anti-γ-H2AX antibody and primary mouse anti-PCNA antibody (Santa Cruz Biotechnology, Santa Cruz, CA) were used as recommended [74]. The γH2AX foci were be counted by eye in a blinded fashion by two independent investigators. At least 400 cells from each studied tissue of each animal were examined [56, 73].

### Western immunoblotting

Western immunoblotting for RAD51, KU70 and POLB was conducted using spleen, thymus, liver, lung, kidney, hippocampus, frontal cortex, cerebellum, olfactory bulb tissue as previously described [75]. Tissue samples were sonicated in 0.4-0.8 ml of ice-chilled 1% sodium dodecyl sulphate (SDS) and boiled for 10 min. Small aliquots (10 μl) of homogenate were reserved for protein determination using protein assay reagents from BioRad (Hercules, CA). Equal amounts of proteins (25 μg) were separated by SDS-polyacrylamide electrophoresis (PAGE) in slab gels of 8 or 12% polyacrylamide, made in triplicates, and transferred to PVDF membranes (Amersham, Baie d'Urfé, Québec). Membranes were incubated with antibodies against RAD51, KU70 (1:1000, BD Biosciences), POLB (1:1000, Biomeda, Foster City, CA). Antibody binding was revealed by incubation with horseradish peroxidase-conjugated secondary antibodies (GE Biosciences) and the ECL Plus immunoblotting detection system (GE Biosciences). Chemiluminescence was detected by Biomax MR films (Eastman Kodak, New Haven, CT). Unaltered PVDF membranes were stained with Coomassie Blue (BioRad, Hercules, CA) and the intensity of the Mr 50,000 protein band was assessed as a loading control. Signals were quantified using NIH ImageJ 1.63 Software and normalized to both actin and the Mr 50,000 protein which gave consistent results.
